# The diagnostic and prognostic value of MRP8/MRP14 in intrahepatic cholangiocarcinoma

**DOI:** 10.18632/oncotarget.5329

**Published:** 2015-10-12

**Authors:** Guang-Zhi Jin, Wei Dong, Hui Dong, Hua Yu, Jia Chen, Wen-Long Yu, Ai-Jun li, Wen-Ming Cong, Meng-Chao Wu

**Affiliations:** ^1^ Department of Pathology, Eastern Hepatobiliary Surgery Hospital, Second Military Medical University, Shanghai 200438, China; ^2^ Department II of Biliary Tract Surgery, Eastern Hepatobiliary Surgery Hospital, Second Military Medical University, Shanghai 200438, China; ^3^ Division of Special Treatment II, Eastern Hepatobiliary Surgery Hospital, Second Military Medical University, Shanghai 200438, China; ^4^ Department of Surgery, Eastern Hepatobiliary Surgery Hospital, Second Military Medical University, Shanghai 200438, China

**Keywords:** myeloid-related protein 8, myeloid-related protein 14, tumor-infiltrating immune cell, intrahepatic cholangiocarcinoma, prognosis

## Abstract

Myeloid-related protein 8 (MRP8) and 14 (MRP14) are abundantly expressed in several kinds of benign and malignant tumors. However, little is known about their clinicopathological significance in intrahepatic cholangiocarcinoma (ICC), biliary intraepithelial neoplasia (BilIN), intraductal papillary neoplasm of bile duct (IPNB), or inflammatory hepatic biliary ducts epithelium (IHBD). This study aimed to investigate the diagnostic and prognostic values of MRP8 and MRP14 as new biomarkers for ICC. We examined MRP8 and MRP14 expression levels by immunohistochemistry in IHBD (*n* = 15), BilIN (BilIN1 = 24, BilIN2 = 9, BilIN3 = 5), IPNB (*n* = 18) and ICC (*n* = 416). The differential diagnostic and prognosis values were also evaluated. The results showed that the ratio of tumor-infiltrating MRP8 and MRP14 positive immune cells, relative to biliary epithelial cells, was significantly increased in ICC tissues compared with nonmalignant tissues, including IHBD, BilIN1, BilIN2, BilIN3, and IPNB (*P* value < 0.05). In addition, over-expression levels of MRP8 and MRP14 were correlated with overall survival (OS) and time to recurrence (TTR) by univariate analysis; MRP8/MRP14 combination was an independent prognostic factor for OS and TTR. MRP8 and MRP14 expression might help to identify the benign bile duct diseases from ICC, as high expression of MRP8 and MRP14 suggests a poor prognosis after surgical resection.

## INTRODUCTION

Intrahepatic cholangiocarcinoma (ICC) is a poorly understood biliary malignancy that accounts for an estimated 10–15% of all primary liver cancers [1] and approximately 8% of cholangiocarcinomas [2]. However, in the United States, the age-adjusted incidence of ICC increased from 0.32 per 100,000 individuals in 1975 to 0.85 per 100,000 individuals in 2000, and is still increasing [3, 4].

Early diagnosis of ICC is possible to carry out radical surgery. Biliary intraepithelial neoplasia (BilIN) and intraductal papillary neoplasm of bile duct (IPNB) are two proposed benign bile duct diseases for the development and progression of ICC. It was reported that the protein expression pattern of mucin core proteins (MUCs)/cytokeratins (CKs) and P16/EZH2/Mmi1 in neoplastic biliary epithelia differed according to the pathway of cholangiocarcinogenesis, via BilIN or IPNB lineage [5, 6].

Partial hepatectomy remains the gold standard of curative treatment for ICC [7, 8]. However, prognosis after partial hepatectomy is unsatisfactory, with a high incidence of locoregional recurrence and/or distant metastases [9–11]. Identifying effective novel prognostic biomarkers that might be related to the development and progression of ICC may help provide new therapeutic strategies.

Myeloid-related protein (MRP) has been implicated in multiple stages of tumorigenesis, progression and prognosis. MRP carries out a wide range of intracellular and extracellular functions, such as regulation of calcium homeostasis, cell proliferation, apoptosis, cell invasion and motility, cytoskeleton interactions, protein phosphorylation, regulation of transcriptional factors, autoimmunity, chemotaxis, inflammation and pluripotency [12].

In this study, we analyzed the involvement of MRP8 and MRP14 in the progression of cholangiocarcinogenesis through BilIN and IPNB, and investigated its role in tumor progression of ICC.

## RESULTS

### MRP8 and MRP14 Expression-level Profiles of IHBD, BilIN1, BilIN2, BilIN3, IPNB, and ICC

**Figure 1:**
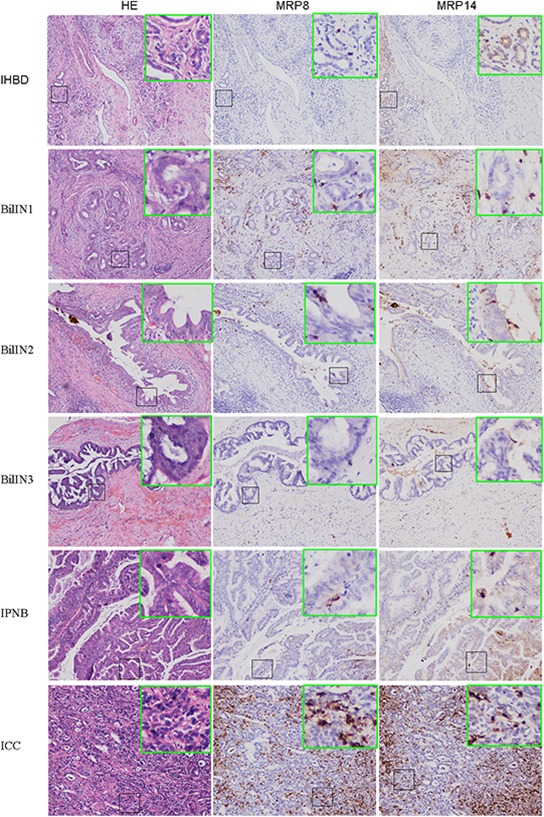
Expression of MRP8 and MRP14 in IHBD, BilIN1, BilIN2, BilIN3, IPNB, ICC H&E staining section shows histopathology of IHBD (A), BilIN1 (D), BinIN2 (G), BinIN3 (J), IPNB (M), ICC (P); MRP8 staining shown in IHBD (B), BilIN1 (E), BinIN2 (H), BinIN3 (K), IPNB (N), ICC (Q); MRP14 staining shown in IHBD (C), BilIN1 (F), BinIN2 (I), BinIN3 (L), IPNB (O), ICC (R) (200× magnification).

**Figure 2:**
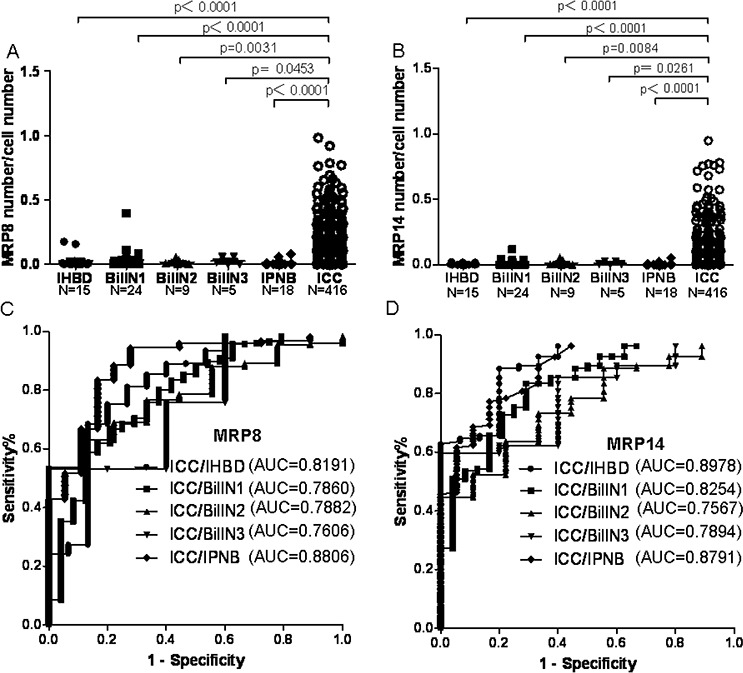
The MRP8 and MRP14 expression level in IHBD, BilIN1, BilIN2, BilIN3, IPNB, ICC and the ROC curve A scatter plot of MRP8 **A.** and MRP14 **B.** positive cells to the total number of biliary epithelial cells was obtained from the TMAs. Mann-Whitney test showed a significant difference between ICC and IHBD, BilIN1, BilIN2, BilIN3, IPNB. ROC curve analyses and AUC showed that MRP8 **C.** and MRP14 **D.** have good performance to distinguish ICC from IHBD, BilIN1, BilIN2, BilIN3, IPNB.

MRP8 and MRP14 were mostly detected in tumor-infiltrating immune cells (neutrophils and monocytes; Supplementary Figure S1) or biliary epithelial cell infiltrating immune cells (Figure 1), as reported previously [13]. The expression level represented by the ratio of infiltrating MRP8 and MRP14 positive cells compared with the number of biliary epithelial cells was calculated. The results showed that the ratio of MRP8 positive cells to biliary epithelial cells in ICC was higher than in IHBD (*P* < 0.0001), BilIN1 (*P* < 0.0001), BilIN2 (*P* = 0.0031), BilIN3 (*P* = 0.0453), and IPNB (*P* < 0.0001). The ratio of MRP14 positive cells to biliary epithelial cells in ICC was higher than in IHBD (*P* < 0.001), BilIN1 (*P* < 0.0001), BilIN2 (*P* = 0.0084), BilIN3 (*P* = 0.0261), and IPNB (*P* < 0.0001) (Figure 2). In addition, the correlation coefficients between MRP8 and MRP14 were *r* = 0.838 (*P* < 0.001) and *r* = 0.823 (*P* < 0.001), respectively (Supplementary Figure S2).

### Diagnostic value

The sensitivity and specificity of the detection of MRP8 are summarized in Supplementary Table S1. The sensitivity and specificity of MRP8 for discriminating ICC from IHBD, BilIN1, BilIN2, BilIN3, and IPNB were 0.7524 and 0.8000, 0.5889 and 0.8750, 0.5361 and 1.0000, 0.5313 and 1.0000, and 0.8341 and 0.8333, respectively. The sensitivity and specificity of MRP14 for discriminating ICC from IHBD, BilIN1, BilIN2, BilIN3, and IPNB were 0.8846 and 0.8000, 0.8341 and 0.7083, 0.6370 and 0.7780, 0.5962 and 1.0000, and 0.7716 and 0.8333, respectively. In addition, discriminating efficacy was represented by area under the curve (AUC) of ROC curves. For MRP8, the AUC was 0.8191 for ICC/IHBD, 0.7860 for ICC/BilIN1, 0.7882 for ICC/BilIN2, 0.7606 for ICC/BilIN3, and 0.8806 for ICC/IPNB. For MRP14, the AUC was 0.8978 for ICC/IHBD, 0.8254 for ICC/BilIN1, 0.7567 for BilIN2, 0.7894 for ICC/BilIN3, and 0.8791 for ICC/IPNB (Figure 2).

### Prognostic significance

In the prognostic group, among 416 patients at the last follow-up, 309 had died and 319 had tumor recurrence. To enhance the prognostic value, the expression level represented by MRP8 and MRP14 positive cells to the biliary epithelial cell number was combined. Kaplan-Meier analysis in both the primary cohort and the testing cohort showed that the overall survival (OS) and time to recurrence (TTR) rates were statistically better in the combined low-MRP group than those in at least one high-MRP group, with the exception of OS in the testing group (*P* = 0.041 for OS and *P* = 0.020 for TTR in primary cohort; *P* = 0.067 for OS and *P* = 0.047 for TTR in testing cohort, Supplementary Figure S3).

**Figure 3:**
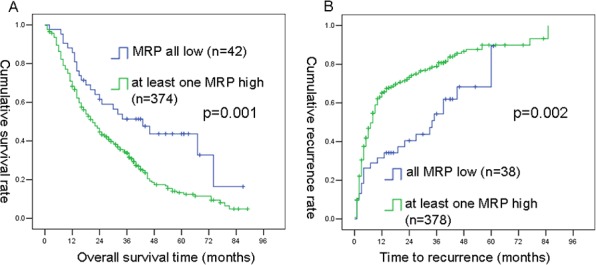
Kaplan-Meier curves of OS and TTR differences among ICC patients in combined primary and testing cohorts MRP8 combined with MRP14 **A.** and **B.** was found to be significant for both OS and TTR (by the log-rank test).

In addition, results from the Kaplan-Meier analyses that combined primary and testing cohorts showed that the OS and TTR rates were significantly better in the combined low-MRP group than those in at least one high-MRP group (*P* = 0.001 for OS, *P* = 0.002 for TTR, Figure 3).

Univariate analysis showed that serum-alpha fetal protein (AFP), -carcino embryonic antigen (CEA), -carbohydrate antigen 199 (CA199), -albumin (ALB), -gamma-glutamyl transferase (GGT), -alanine transaminase (ALT), -alkaline phosphatase (ALP), tumor size, tumor number, micro-vascular invasion, tumor-nodes-metastases (TNM), MRP8 number, MRP8 ratio, MRP14 number, MRP8 ratio, and combined MRP8/MRP14 ratio were predictors for OS in a combined primary and testing cohort. Furthermore, liver cirrhosis, serum-CEA, -CA199, -GGT, -ALP, tumor size, tumor number, micro-vascular invasion, TNM, MRP8 number, MRP8 ratio, MRP14 number, MRP14 ratio, and MRP8/MRP14 ratio combination were predictors for TTR in combined primary and testing cohorts. Multivariate Cox regression analysis indicated that serum-AFP, -CEA, -ALB, -ALP, tumor size, tumor number, TNM, and MRP8 ratio were independent prognostic factors for OS in ICC patients after the surgical resection. In addition, liver cirrhosis, serum-CEA, -ALP, tumor size, tumor number, TNM, and MRP8/MRP14 ratio combination were independent prognostic factors for TTR in ICC patients after the surgical resection in combined primary and testing cohort (Table 1). Similarly, univariate analysis in the primary cohort showed that serum-CEA, -CA199, -ALB, -GGT, -ALT, -ALP, tumor size, TNM, and MRP8/MRP14 ratio combination were predictors for OS; serum-ALB, -ALP, tumor size, TNM, and MRP8/MRP14 ratio combination were predictors for TTR (Supplementary Table S2). In the testing cohort, serum-AFP, -CEA, -CA199, -ALB, -GGT, -ALP, tumor size, tumor number, micro-vascular invasion, TNM, and MRP8 number were predictors for OS; liver cirrhosis, serum-CEA, -CA199, -GGT, -ALP, tumor size, tumor number, micro-vascular invasion, TNM, MRP8 ratio, MRP8 number, MRP14 ratio were predictors for TTR (Supplementary Table S3).

**Table 1:** Univariate and multivariate analyses of factors associated with OS and TTR in combined primary and testing cohorts

Factors	OS				TTR			
	Univariate *p*	HR	Multivariate 95% Cl	*p*	Univariate *p*	HR	Multivariate 95% Cl	*p*
Age: ≤53 *vs.* >53	0.719				0.100			
Sex: male *vs.* female	0.946				0.310			
Liver cirrhosis: no *vs*. yes	0.360				**0.014**	0.732	0.558–0.961	**0.024**
HBsAg: negative *vs*. positive	0.071				0.079			
Serum AFP, μg/L: ≤low *vs*. >high	**<0.0001**	1.336	1.039–1.717	**0.024**	0.054			
Serum CEA, μg/L: ≤low *vs*. >high	**<0.0001**	1.696	1.272–2.262	**<0.0001**	**0.001**	1.596	1.138–2.235	**0.007**
Serum CA199, U/ml: ≤low *vs*. >high	**<0.0001**				**0.010**			
Serum ALB, g/L: ≤low *vs*. >high	**<0.0001**	0.565	0.413–0.773	**<0.0001**	0.236			
Serum GGT, U/L: ≤low *vs*. >high	**<0.0001**				**0.005**			
Serum ALT, U/L: ≤low *vs*. >high	**0.038**				0.247			
Serum ALP, U/L: ≤low *vs*. >high	**<0.0001**				**<0.0001**	1.462	1.153–1.853	**0.002**
Tumor size, cm: ≤low *vs*. >high	**<0.0001**	1.734	1.300–2.313	**<0.0001**	**<0.0001**	1.752	1.222–2.511	**0.002**
Tumor number: single *vs*. multiple	**<0.0001**	1.499	1.133–1.983	**0.005**	**<0.0001**	1.563	1.185–2.063	**0.002**
Micro-vascular invasion: no *vs*. yes	**<0.0001**				**0.028**			
TNM: I vs. II *vs*. III *vs.* IV	**<0.0001**	1.200	1.067–1.350	**0.002**	**<0.0001**	1.130	1.008–1.268	**0.036**
MRP8 number: ≤low *vs*. >high	**0.021**				**0.021**			
MRP8 ratio: ≤low *vs*. >high	**0.007**	1.584	1.076–2.333	**0.020**	**0.012**			
MRP14 number: ≤low *vs*. >high	**0.005**				**0.005**			
MRP14 ratio: ≤low *vs*. >high	**0.003**				**0.005**			
MRP8 ratio/MRP14 ratio combination: all low vs. at least one high	**0.002**				**0.002**	1.740	1.137–2.662	**0.011**

**Note:** OS, overall survival; TTR, time to recurrence; Cl, confidence interval; HBsAg, hepatitis B surface antigen; AFP, alpha-fetoprotein; CEA, carcino embryonic antigen; CA199, carbohydrate antigen 19-9; ALB, albumin; GGT, gamma-glutamyl transpeptidase; ALP, alkaline phosphatase; TB, total bilirubin; ALT, alanine transaminase; TNM, tumor-nodes-metastases.

## DISCUSSION

ICC is a unique liver cancer that differs from hepatocellular carcinoma (HCC) [14], hilar and distal bile duct cholangiocarcinoma [15] because of its different mechanisms of carcinogenesis, biologic behaviors, clinical features, imaging manifestations and therapeutic strategies. Despite great clinical achievements in the treatment of ICC during the past few decades, the prognosis of patients with ICC is still unsatisfactory, with a high incidence of locoregional recurrence and/or distant metastases [9–11]. Unfortunately, there are limited prognostic markers for ICC, and the disease requires a distinct prognostic predictive model.

Improving the survival rate of patients with a malignant tumor after surgical resection requires clinicians to engage in the active treatment of recurrence and investigate the biological and clinicopathological characteristics that reflect tumor behavior, such as progressive or metastatic capability [16, 17].

MRP8 and MRP14 belong to the S100 gene family, which is the largest subfamily of EF-hand calcium-binding proteins. At least 25 distinct members of this subgroup have been described [18]. S100 proteins form either homodimeric or heterodimeric complexes with one another [19, 20] and have a wide range of intracellular and extracellular functions. Intracellular functions include: regulation of calcium homeostasis, cell cycle, cell growth and migration, phosphorylation, cytoskeletal components and transcriptional factors. In contrast to their intracellular function, extracellular S100 proteins act in a cytokine-like manner by binding to cell surface receptors, such as the receptor for advanced glycation end products (RAGE) and toll-like receptors (TLRs) [18, 21].

There is increasing interest in the S100 proteins and their relationship with inflammatory bile duct diseases [22], BilIN and ICC [23], as well as both malignant and benign gallbladder diseases [24], because of their altered expression in various malignancies. In a recent study, the prognostic significance of S100A4 in ICC was evaluated [25]. However, the report did not address the diagnostic or prognostic role of MRP8 or MRP14 in IHBD, BilIN, IPNB, and ICC. Despite the statistical power analysis (Supplementary Table S4) showed that it may be need to more large scale validation, however, it is still a first observation of diagnostic role of MRP8 and MRP14 in ICC and benign biliary tissues.

In this study, the diagnostic and prognostic values of MRP8 and MRP14 in BilIN, IPNB, and ICC were investigated. We show, for the first time, that MRP8 and MRP14 are significantly increased in infiltrating immune cells of ICC tissues, compared with other nonmalignant tissues, such as IHBD, BilIN1, BilIN2, BilIN3, and IPNB. Moreover, high expression levels of MRP8 and MRP14 were indicative of a poor prognosis for ICC patients after surgical resection.

In addition, we introduced a novel scoring method for evaluating the MRP protein expression level, which is represented by MRP positive cells/biliary epithelial cell number. Furthermore, X-Tile plots were created for assessment of a best cutoff point for continuous variables and optimized cutoff points based on outcome. Our results found novel cutoff points, such as serum-AFP of 7.5 ng/ml, -CEA of 5.2 ng/ml, -CA199 of 40 U/L, -ALB of 37.3 mg/ml, -GGT of 54 U/L, -ALT of 50 U/L, -ALP of 113 U/L, tumor size of 4 cm, MRP8 number of 8, MRP8 ratio of 0.0498, MRP14 number of 5, and MRP8 ratio of 0.0025. Thus the novel assay method and cutoff points proposed in this study may significantly enhance the inter-laboratory assay and intra-laboratory assay reproducibility.

Accumulating evidence shows that stromal cells in neoplastic tissues play central roles in tumor progression and cancer metastasis [26]. These stromal cells include fibroblasts, vascular cells, and infiltrating leukocytes, as well as bone marrow-derived myeloid cells, including macrophages, neutrophils, mast cells, myeloid cell-derived suppressor cells and mesenchymal stem cells [27]. In addition, MRP8 and MRP14 proteins are highly expressed in tumor-infiltrating myeloid cells in many epithelial tumors. Therefore, MRP8 and MRP14 proteins are important constituents of the micro-environment that critically contributes to the development of tumors [28, 29]. Our study has shown, for the first time, that MRP8 and MRP14 protein expressions are significantly increased in ICC tissues and correlate with the prognosis of ICC patient after surgical resection.

In conclusion, the expressions of MRP8 and MRP14 were significantly increased in infiltrating immune cells of ICC tissues compared with IHBD, BilIN1, BilIN2, BilIN3, and IPNB. The numbers of MRP8 and MRP14 positive cells, compared with the total number of biliary epithelial cells, are useful markers for predicting the progression and prognosis of ICC. MRP8 might have a synergistic effect with MRP14 in predicting the prognosis of ICC patients after surgical resection.

## MATERIALS AND METHODS

### Patients

Four hundred and sixteen ICC specimens were surgically resected and diagnosed at the Eastern Hepatobiliary Surgery Hospital from July 2000 to December 2008, among which 207 cases comprised the primary cohort and 209 cases comprised the testing cohort. Biliary lesions were surgically resected and diagnosed at the same hospital from March 2008 to September 2013. The biliary lesions were divided into intrahepatic biliary epithelium (IHBD) with inflammation (*n* = 18), BilIN1 (*n* = 25), BilIN2 (*n* = 12), BilIN3 (*n* = 5), and IPNB (*n* = 19) (Supplementary Table S5). Hematoxylin and eosin (H&E)-stained slides made from each formalin-fixed paraffin-embedded tissue that had undergone surgical resection were reviewed by two experienced hepatopathologists (WM Cong and H Dong). IHBD was defined as the mildly hyperplastic epithelium with intraepithelial neutrophils and lymphocytic infiltration, lacking a definite disturbance of cell polarity [23]. The histopathologic definition of BilIN was based on the classification proposed by Zen et al. [30]. Based on atypia, BilIN was classified into three grades: BilIN-1 (low-grade lesion), BilIN-2 (high-grade lesion), and BilIN-3 (carcinoma in situ). Liver cirrhosis was conformed pathologically. The definition of ICC and IPNB was based on the classification proposed by the World Health Organization [31].

### Follow-up

For prognostic evaluation, the follow-up period of all 416 ICC patients lasted until September 2014 and complete follow-up data for patients in the prognostic group were available. The overall survival (OS) was defined as the length of time between surgery and death, or the last follow-up examination. The time to recurrence (TTR) was calculated from the date of tumor resection until the detection of tumor recurrence, death or last observation. Detailed follow-up procedures are described in the Supplementary Data. The median OS time was 27 months (range 0.1–89 months) and the median TTR time was 7 months (range 0.1–85 months).

### Tissue microarrays, immunohistochemistry and scoring

Tissue microarrays, immunohistochemistry and scoring of all specimens were selected randomly and tissue microarrays were constructed from two representative cores from each specimen. Immunohistochemistry was performed and samples were measured as reported previously [32]. Briefly, H&E-stained 4-μm-thick sections were placed on slides coated with 3-aminopropyltriethoxysilane. Paraffin sections were deparaffinized in xylene and rehydrated through decreasing concentrations of ethanol (100%, 95%, and 85%, 5 min each). Antigens were retrieved by microwave irradiation for 3 min in pH 6.0 citric buffer and cooled at room temperature for 60 min. Endogenous peroxidase activity was blocked by incubation of the slides in 3% H_2_O_2_/phosphate-buffered saline, and nonspecific binding sites were blocked with goat serum. Primary antibodies were diluted as follows: rabbit monoclonal antibody against MRP8 (ab 92331; Abcam; Clone ID: EPR3554, 1/5000 dilution); rabbit polyclonal antibody against calgranulin B (S100A9/MRP14) (sc-20173; Santa Cruz; 1/2500 dilution). The expression levels of MRP8 and MRP 14 were represented by the ratio of number of positive cells to the biliary epithelial cell number.

The imaging system comprised a Leica CCD camera, DFC420, connected to a Leica DM IRE2 microscope (Leica Microsystems Imaging Solutions, Cambridge, UK). All samples had one field captured from each core under high-power magnification (200 ×) using Leica QW in Plus V3 software. Therefore, a total of two photographs from each sample were used to count the cell number using Image-Pro Plus v6.0 software (Media Cybernetics, Bethesda, MD. USA). Next, the ratio (relative positive numbers) of MRP8 and MRP 14 positive cells (absolute positive numbers) to the total number of biliary epithelial cells was calculated [33]. Finally, the mean ratios were calculated from two photographs and used as the ratio of specimen.

### Statistical analysis

Statistical analyses were carried out with SPSS 13.0 software (SPSS, Chicago, IL, USA) and GraphPad Prism 5.01. The significance of expression level for markers between ICC, IHBD and neoplasia was analyzed by the Mann-Whitney test. To evaluate the discriminating efficacy, receiver operating characteristic (ROC) analysis was performed. For calculating the best cut-off points for OS and TTR, the X-Tile statistical package (version 3.5.0, Yale University, New Haven, CT, USA) was used. X-tile plot illustrates the presence of substantial tumor subpopulations and shows the robustness of the relationship between a biomarker and outcome by construction of a two dimensional projection of every possible subpopulation [34]. X-tile plots were created for assessment of level of quantitative factors including serum AFP, -CEA, -CA199, -ALB, -GGT, -ALT, -ALP, tumor size, MRP8 number, MRP8 ratio, MRP14 number, MRP14 ratio. Statistical significance was assessed using the cut-off points derived from 416 cases by a standard log-rank method, with *P* values obtained from a lookup table (each cut-off and *p* values for OS and TTR was showed in Supplementary Table S6). Univariate and multivariate Cox regression analyses were performed to determine the predictors of ICC. All statistical tests were two-sided, and a *P* value of < 0.05 was considered statistically significant. Kaplan-Meier analysis and the log-rank test were used to compare the time of categorization of continent patients. G Power (version Win3.1.9.2) was used to perform the statistical power analyses [35].

## SUPPLEMENTARY FIGURES AND TABLES


