# Supplementary material for: The diagnostic and prognostic value of MRP8/MRP14 in intrahepatic cholangiocarcinoma
Source: Oncotarget. 2015 Oct 12;6(36):39357–64. doi: 10.18632/oncotarget.5329 (PMC4770777; doi:10.18632/oncotarget.5329)
Supplement: Supplementary file 1 [file oncotarget-06-39357-s001.pdf]

SUPPLEMENTARY FIGURES AND TABLES

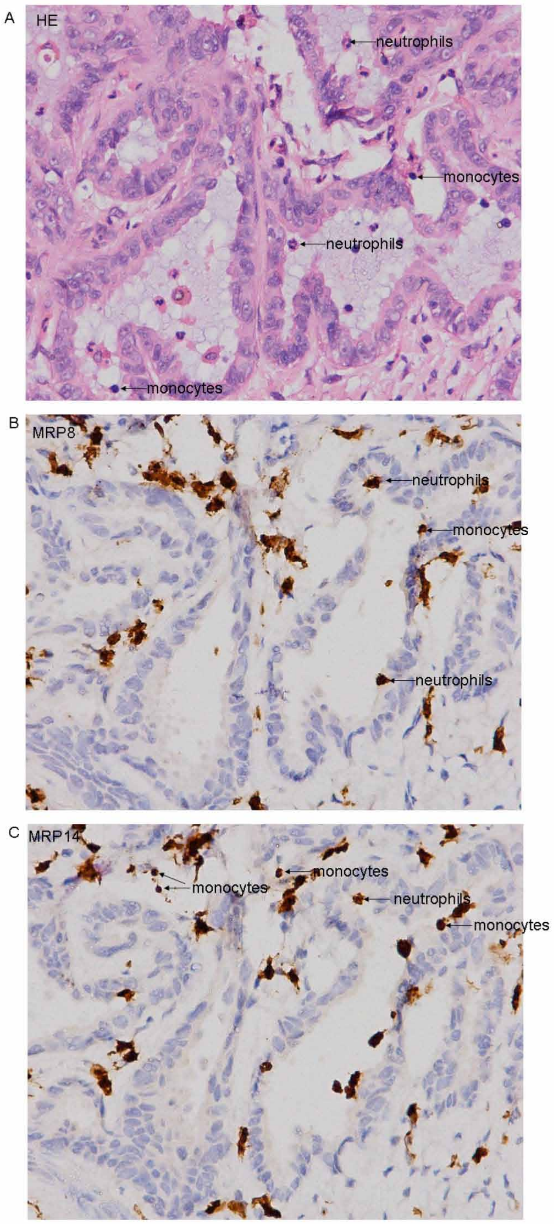

Supplementary Figure S1: MRP8 expression in tumor-infiltrating immune cells or biliary epithelial cells-infiltrating immune cells. Typical HE-stained sections **A.** and strong immunostaining for MRP8 **B.** and MRP14 **C.** was mostly detected in tumor-infiltrating immune cells, arrowhead for representive monocytes and neutrophils. (×400 magnification).

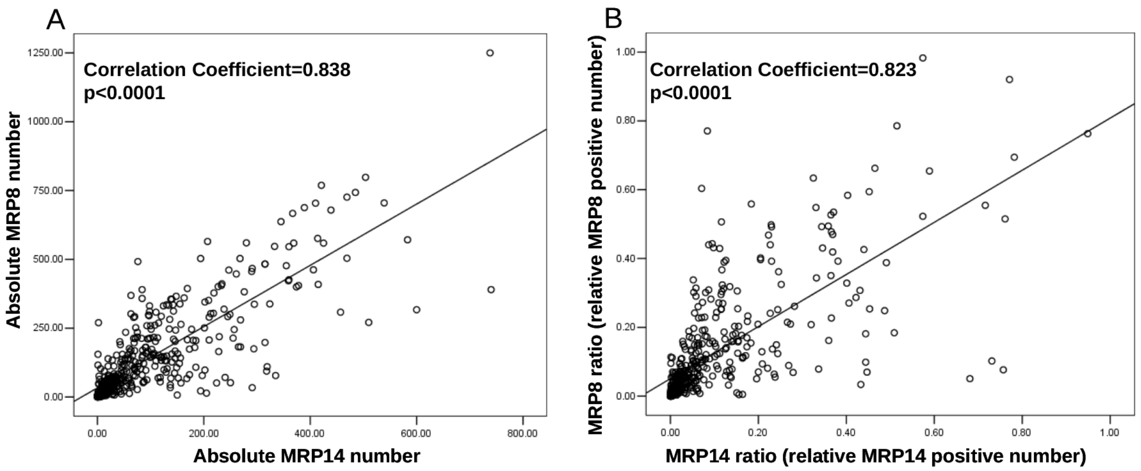

Supplementary Figure S2: Correlation between MRP8 and MPR14 expression in ICC. Significance was estimated by use of the person correlation.

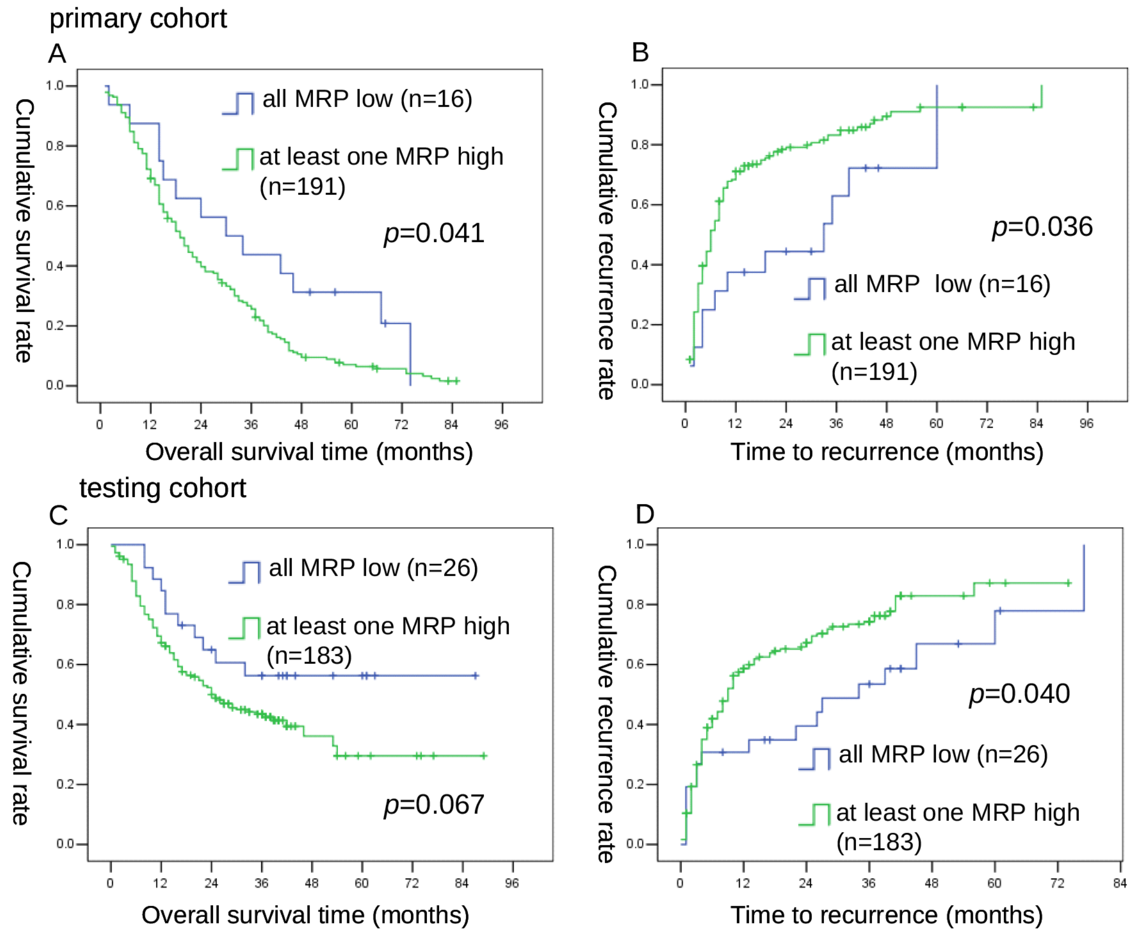

Supplementary Figure S3: Kaplan-Meier curves of OS and TTR differences among ICC patients in primary cohort A. and B. and in testing cohort C. and D. Expression of combination of MRP8 and MRP14 were significant by the Log-rank test for both OS and TTR.

Supplementary Table S1: Sensitivity, specificity, cut off point, positive and negative predictive values for ICC detection of MRP8 and MRP14 expression

	MRP8 positive cell/biliary epithelial cell			MRP14 positive cell/biliary epithelial cell		
	Sensitivity	Specificity	cutoff point	Sensitivity	Specificity	cutoff point
ICC/IHBD	0.7524	0.8000	0.0174	0.8846	0.8000	0.0040
ICC/BilIN1	0.5889	0.8750	0.0464	0.8341	0.7083	0.0059
ICC/BilIN2	0.5361	1.0000	0.0561	0.6370	0.7780	0.0206
ICC/BilIN3	0.5313	1.0000	0.0548	0.5962	1.0000	0.0267
ICC/IPNB	0.8341	0.8333	0.0101	0.7716	0.8333	0.0085

Note: IHBD, inflammatory hepatic biliary ducts epithelium; BilIN, biliary intraepithelial neoplasia; IPNB, intraductal papillary neoplasm of bile duct, ICC, intrahepatic cholangiocarcinoma.

Supplementary Table S2: Univariate and multivariate analyses of factors associated with OS and TTR in primary cohort

Factors	OS				TTR			
	univariate *p*	HR	multivariate 95% Cl	*p*	univariate *p*	HR	multivariate 95% Cl	*p*
Age: ≤53 *vs.* >53	0.561				0.552			
Sex: male *vs.* female	0.142				0.157			
Liver cirrhosis: no *vs.* yes	0.522				0.324			
HBsAg: negative *vs.* positive	0.156				0.286			
Serum AFP, µg/L: ≤low *vs.* >high	0.762				0.332			
Serum CEA, µg/L: ≤low *vs.* >high	**<0.0001**	1.750	1.231–2.487	**0.002**	0.106			
Serum CA199, U/ml: ≤low *vs.* >high	**<0.0001**				0.796			
Serum ALB, g/L: ≤low *vs.* >high	**0.004**	0.524	0.345–0.797	**0.003**	**0.036**	0.639	0.411–0.993	**0.047**
Serum GGT, U/L: ≤low *vs.* >high	**0.009**				0.051			
Serum ALT, U/L: ≤low *vs.* >high	**0.033**				0.611			
Serum ALP, U/L: ≤low *vs.* >high	**0.004**				**0.012**			
Tumor size, cm: ≤low *vs.* >high	**0.004**	1.466	1.020–2.106	**0.039**	**0.009**	1.728	1.097–2.721	**0.018**
Tumor number: single *vs.* multiple	0.109				0.076			
Micro-vascular invasion: no *vs.* yes	0.098				0.581.			
TNM: I vs. II *vs.* III *vs.* IV	**<0.0001**	1.222	1.068–1.399	**0.004**	**0.005**	1.187	1.024–1.375	**0.023**
MRP8 number: ≤low *vs.* >high	0.158				0.194			
MRP8 ratio: ≤low *vs.* >high	0.099				0.172			
MRP14 number: ≤low *vs.* >high	0.087				0.067			
MRP14 ratio: ≤low *vs.* >high	0.065				0.035			
MRP8 ratio/MRP14 ratio combination: all low vs. at least one high	**0.049**				**0.028**			

Note: Univariate analysis was calculated by the Cox proportional hazards regression model. Multivariate analysis was performed using the Cox multivariate proportional hazard regression model with stepwise manner (forward, likelihood ratio). HBsAg, hepatitis B surface antigen; AFP, alpha-fetoprotein; CEA, carcino embryonic antigen; CA199, carbohydrate antigen 19-9; ALB, Albumin; GGT, gamma -glutamyl transpeptadase; ALP, alkaline phosphatase; TB, total bilirubin; ALT, alanine transaminase; TNM, tumor-nodes-metastases.

Supplementary Table S3: Univariate and multivariate analyses of factors associated with OS and TTR in testing cohort

Factors	OS				TTR			
	univariate *p*	HR	multivariate 95% Cl	*p*	univariate *p*	HR	multivariate 95% Cl	*p*
Age: ≤53 *vs*. >53	0.486				0.099			
Sex: male *vs*. female	0.379				0.968			
Liver cirrhosis: no *vs*. yes	0.353				**0.004**			
HBsAg: negative *vs*. positive	0.238				0.125			
Serum AFP, µg/L: ≤low *vs*. >high	**0.022**				0.322			
Serum CEA, µg/L: ≤low *vs*. >high	**0.022**				**0.003**			
Serum CA199, U/ml: ≤low *vs*. >high	**0.001**	1.559	1.044–2.397	**0.030**	**0.001**			
Serum ALB, g/L: ≤low *vs*. >high	**0.001**				0.891			
Serum GGT, U/L: ≤low *vs*. >high	**0.001**				**0.042**			
Serum ALT, U/L: ≤low *vs*. >high	0.536				0.345			
Serum ALP, U/L: ≤low *vs*. >high	**0.005**				**0.002**			
Tumor size, cm: ≤low *vs*. >high	**<0.0001**	2.092	1.297–3.373	**0.002**	**<0.0001**	2.790	1.571–4.954	**<0.0001**
Tumor number: single *vs*. multiple	**<0.0001**	1.753	1.087–2.826	**0.021**	**<0.0001**	2.835	1.855–4.333	**<0.0001**
Micro-vascular invasion: no *vs*. yes	**0.001**	1.652	1.016–2.686	**0.043**	**0.014**			
TNM: I vs. II *vs*. III *vs*. IV	**<0.0001**	1.256	1.018–1.551	**0.033**	**0.002**			
MRP8 number: ≤low *vs*. >high	0.015				0.079			
MRP8 ratio: ≤low *vs*. >high	0.182				**0.034**	1.584	1.097–2.533	**0.017**
MRP14 number: ≤low *vs*. >high	0.051				**0.030**			
MRP14 ratio: ≤low *vs*. >high	0.074				**0.048**			
MRP8 ratio/MRP14 ratio combination :all low *vs*. at least one high	0.074				0.057			

Note: Univariate analysis was calculated by the Cox proportional hazards regression model. Multivariate analysis was performed using the Cox multivariate proportional hazard regression model with stepwise manner (forward, likelihood ratio). HBsAg, hepatitis B surface antigen; AFP, alpha-fetoprotein; CEA, carcino embryonic antigen; CA199, carbohydrate antigen 19-9; ALB, Albumin; GGT, gamma -glutamyl transpeptadase; ALP, alkaline phosphatase; TB, total bilirubin; ALT, alanine transaminase; TNM, tumor-nodes-metastase.

Supplementary Table S4: Statistical power analyses

Parameters	IHBD/ICC	BillN 1/ICC	BillN 2/ICC	BillN 3/ICC	IPNB/ICC
noncentrality parameter δ	1.902	2.382	1.484	1.111	2.077
critical t	1.965	1.965	1.965	1.965	1.965
Df	429	438	423	419	432
power (1-β err prob)	0.475	0.662	0.316	0.198	0.545

Note: test type: *t* test; statistical test type:Means:difference between two indepentent means (two groups); type of power analysis:Post hoc:compute achieved power-given α, sample size and effect size; tail: two; effect size d:0.5; α err prob:0.05; sample size: ICC = 416, IHBD = 15, BillN 1=15, BillN 2 = 9, BillN 3 = 5, IPNB = 18.

Supplementary Table S5: Demographics and Clinicopathologic Characteristics of Patients

factor	IHBD (*n* = 15)	BilIN (*n* = 38)	IPNB (*n* = 18)	ICC (*n* = 416)
age				
median	50	54.5	58.5	52.5
range	26–54	25–73	45–77	26–80
sex				
male	7	15	10	302
female	8	23	8	114
serum HBsAg				
negative	11	32	15	188
positive	4	6	3	228
serum AFP				
median	2.2	2.6	2.5	4.6
range	1.4–701	0.9–287.9	1.4–8.5	0–66210
serum CEA				
median	1.6	2.6	3	2
range	0.4–17	0.6–51.9	0.3–10.2	0–35134
serum CA199				
median	17.7	17.9	25.2	31
range	0.6–438	0.6–1000.0	0.6–647.5	0–12000
serum ALB				
median	41.2	41.1	40.8	42.1
range	36.3–50.0	29.8–50.7	30.5–46.7	26.6–52.2
serum GGT				
median	91	159	161.5	77
range	14.0–1159.0	9.0–1073.0	23.0–1101.0	9–2029
serum ALP				
median	61	89.5	140	102
range	14.6–402	25–542.0	58.0–559.0	4.6–947
serumTB				
median	15.7	12.8	13	13.3
range	5.9–64.1	6.3–215.5	6.8–206.2	4–470
serum ALT				
median	32	35	33.5	31
range	12.9–256	6.3–222.6	9.0–428.0	3.5–554
MRP8number				
median	4	4.8	5.5	76.8
range	0–30	0–141	0–155.5	0–1250

factor	IHBD (*n* = 15)	BilIN (*n* = 38)	IPNB (*n* = 18)	ICC (*n* = 416)
MRP8ratio				
median	0.0089	0.0063	0.0025	0.0625
range	0–0.1760	0–0.3970	0–0.081	0–0.98296
MRP14number				
median	0.500	1	1	57.3
range	0–8.5	0–35	0–44	0–740
MRP14ratio				
median	0.0026	0.0021	0.00086	0.04714
range	0.0–0.021	0.0–0.118	0.0–0.053	0–0.9485
tumor size	-	-	-	
median				6
range				0.6–27
tumorNumber				
single	-	-	-	329
multiple				87
microvascular invasion	-	-	-	
no				326
yes				90
TNM	-	-	-	
I				200
II				147
III				7
IV				63

Note: IHBD, inflammatory hepatic biliary ducts epithelium; BilIN, biliary intraepithelial neoplasia; IPNB, intraductal papillary neoplasm of bile duct, ICC, intrahepatic cholangiocarcinoma (ICC); HBsAg, hepatitis B surface antigen; AFP, alpha-fetoprotein; CEA, carcino embryonic antigen; CA199, carbohydrate antigen 19–9; ALB, Albumin; GGT, gamma -glutamyl transpeptadase; ALP, alkaline phosphatase; TB, total bilirubin; ALT, alanine transaminase; TNM, tumor-nodes-metastases.

Supplementary Table S6: Cuf-off points and *p* values for OS and TTR from x-tile analyses

Factors	OS cutoff	*P* value	TTR cutoff	*P* value
serum AFP	3.3	0.0130	3.0	0.4470
serum CEA	5.1	0.0008	11.5	0.0130
serum CA199	79.0	<0.0001	80.0	0.1116
serum ALB	37.3	0.0026	no	no
serum GGT	54.0	0.0008	32.0	0.0660
serum ALT	42.9	0.3841	no	no
serum ALP	97.0	<0.0001	113.0	0.0005
tumor size	4.0	<0.0001	3.0	<0.0001
MRP8 number	8.0	0.2482	11.0	0.2288
MRP8 ratio	0.049755	0.1116	0.012733	0.1325
MRP14 number	5	0.0860	4.0	0.0721
MRP14 ratio	0.002549	0.0579	0.002702	0.0660

Follow-up procedures

Patients with ICC who underwent curative resection between Jul 2000 to Dec 2008 (primary cohort: Jul 2000 to Mar 2006, $n = 207$; testing cohort: Mar 2006 to Dec 2008, $n = 209$) at the Eastern Hepatobiliary Surgery Hospital, Second Military, China. Patients were observed once every 2 months in the first 2 years after surgery and then every 3 to 6 months thereafter. Blood was taken for serum CA19-9, -CEA, -α-fetoprotein, and liver function tests, and an abdominal ultrasound was carried out. Contrast-enhanced CT or magnetic resonance imaging was performed once every 6 months or earlier when tumor recurrence or metastasis was suspected. Further investigation was carried out when clinically indicated. ICC recurrence/metastasis was defined as the appearance of a newly detected tumor confirmed on two radiologic images, with or without elevation of serum tumor markers. The overall survival (OS) was defined as the length of time between surgery and death, or the last follow-up examination. The time to recurrence (TTR) was calculated from the date of tumor resection until the detection of tumor recurrence, death or last observation.
